# A novel DNA-binding protein modulating methicillin resistance in *Staphylococcus aureus*

**DOI:** 10.1186/1471-2180-9-15

**Published:** 2009-01-27

**Authors:** Miriam Ender, Brigitte Berger-Bächi, Nadine McCallum

**Affiliations:** 1 Institute of Medical Microbiology, Gloriastr. 32, CH8006 Zurich, University of Zurich, Zurich, Switzerland

## Abstract

**Background:**

Methicillin resistance in *Staphylococcus aureus *is conferred by the *mecA*-encoded penicillin-binding protein PBP2a. Additional genomic factors are also known to influence resistance levels in strain specific ways, although little is known about their contribution to resistance phenotypes in clinical isolates. Here we searched for novel proteins binding to the *mec *operator, in an attempt to identify new factor(s) controlling methicillin resistance phenotypes.

**Results:**

Analysis of proteins binding to a DNA fragment containing the *mec *operator region identified a novel, putative helix-turn-helix DNA-binding protein, SA1665. Nonpolar deletion of SA1665, in heterogeneously methicillin resistant *S. aureus *(MRSA) of different genetic backgrounds, increased methicillin resistance levels in a strain dependent manner. This phenotype could be fully complemented by reintroducing SA1665 in trans. Northern and Western blot analyses, however, revealed that SA1665 had no visible influence on *mecA *transcription or amounts of PBP2a produced.

**Conclusion:**

SA1665 is a new chromosomal factor which influences methicillin resistance in MRSA. Although SA1665 bound to the *mecA *promoter region, it had no apparent influence on *mecA *transcription or translation, suggesting that this predicted DNA-binding protein modulates resistance indirectly, most likely through the control of other genomic factors which contribute to resistance.

## Background

Methicillin resistant *S. aureus *(MRSA) are an ever increasing threat, both in clinical settings and more recently as an emerging community acquired pathogen. Their invasiveness and pathogenesis relies on a variable arsenal of virulence factors, paired with resistance to virtually all β-lactams and their derivatives. Their ability to rapidly generate resistance to other unrelated classes of antibiotics, or to take up additional resistance determinants, severely hampers therapy and eradication.

In *S. aureus*, methicillin resistance is conferred by an acquired, β-lactam-insensitive penicillin-binding protein (PBP), PBP2a [1-4]. PBP2a is encoded by *mecA*, which is divergently transcribed from its cognate regulators, *mecR1 *(sensor/signal transducer) and *mecI *(repressor). If *mecR1-mecI *are absent or truncated, transcriptional control of *mecA *is taken over by the structurally similar *blaZ *(penicillinase) regulatory elements *blaR1*/*blaI*, if present. In the absence of both regulatory loci, *mecA *is constitutively transcribed [5,6]. In the presence of β-lactams, the transmembrane sensor/signal transducers BlaR1/MecR1, undergo a conformational change, followed by autoproteolytic cleavage of the n-terminal cytoplasmic domain, leading to the activation of the cytoplasmic peptidase and subsequent dissociation of the repressor due to proteolytic degradation [7-9]. However, the signal transduction cascade of this regulatory system has still not been completely elucidated.

Oxacillin resistance levels conferred by *mecA *are strain specific and can vary greatly, with oxacillin minimal inhibitory concentrations (MICs) of different strains ranging from phenotypically susceptible levels, as low as 1 μg/ml up to extremely high values of > 500 μg/ml. Methicillin resistance is also generally expressed heterogeneously. Heterogeneously resistant MRSA, when exposed to β-lactam antibiotics, segregate highly resistant subpopulations, which are much more resistant than the majority of the cells [10]. The frequency of highly resistant subclones generated is often well above the spontaneous mutation frequency, and once selected high level resistance often remains stable, even in the absence of selective pressure. There is currently no satisfactory genetic model which explains how these higher resistance levels are triggered or selected and exactly what factors are functionally responsible for the increased resistance in clinical isolates. Methicillin resistance levels are known to not directly correlate with *mecA *transcription or levels of PBP2a produced [11,12]. However, resistance levels can be manipulated by environmental conditions, such as temperature, pH, osmolarity, and medium composition [13,14].

It has been shown experimentally, that in addition to *mecA*, methicillin resistance depends on the correct interplay of a multitude of genomic factors, termed *fem/aux *factors, including genes involved in peptidoglycan precursor formation, composition and turnover; teichoic acid synthesis; and genes of unknown or poorly characterised functions [15-18]. In addition to structural genes, many regulatory loci have also been shown to influence resistance levels, including global regulators of virulence factor production such as the quorum sensing *agr *system, the staphylococcal accessory regulator SarA and the alternate sigma factor σ^B ^[19,20]; regulators of metabolism, such as the catabolite control protein A (CcpA) [21]; and the VraSR two-component sensor transducer, which induces the cell wall stress stimulon in response to cell wall active antibiotic challenge [22].

The vast MIC differences between MRSA strains, the population heterogeneity within single strains and the dependence of resistance levels on external factors are reflected in these many structural genes and global regulators, which can influence resistance levels.

While typically considered nosocomial pathogens, new faster growing and apparently more virulent MRSA have begun spreading in the community. Interestingly, these emerging strains often express very low methicillin resistance, e.g. the MRSA clone spreading amongst intravenous drug users in the Zurich area, which has an in vitro doubling time of 25 min, but oxacillin MICs of only 0.5 to 4 μg/ml [23]. This particular clone's low-level resistance is partially due to a promoter mutation, leading to tight repression of *mecA*, but resistance levels appear to be mainly restricted by unknown factors within its genomic background [12].

To identify potential factors involved in *mecA *regulation or methicillin resistance levels in such an extremely low level resistant MRSA, we performed DNA-binding protein purification assays, using the *mecA *operator region as bait. A novel, uncharacterized protein, SA1665, was found to bind to this DNA fragment, and shown to increase methicillin resistance levels when deleted.

## Results

### Identification of SA1665

MRSA strain CHE482 is the type strain for the so-called "drug clone" spreading amongst intravenous drug users in the Zurich area [12,23]. This strain carries *mecA *and expresses PBP2a, but appears phenotypically methicillin susceptible by conventional phenotypic tests. However, like most other low-level resistant MRSA, it can segregate a small proportion of higher resistant subclones in the presence of β-lactams. We hypothesized that regulation of methicillin resistance in such low-level resistant clonal lineages may differ qualitatively from classical heterogeneously- or highly-resistant MRSA.

**Figure 1:**
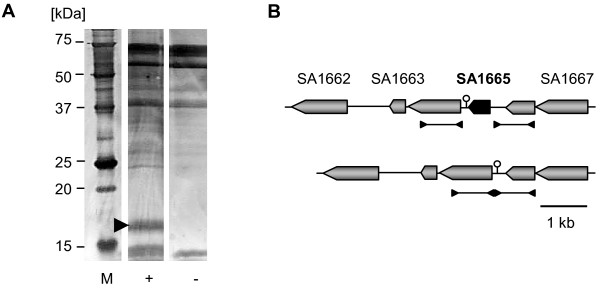
**DNA-binding protein purification assay using *mec *operator DNA region as a bait**. A, Silver stained SDS-polyacrylamide protein gel containing the elutions from DNA-binding protein capture assays performed with either DNA-coated (+) or uncoated (-) streptavidin magnetic beads. One protein band, indicated by the arrow, was only captured by the DNA-coated beads, indicating that it bound specifically to the *mec *operator DNA. The protein size marker (M) is shown on the left. B, Organisation of the genomic region surrounding SA1665. The regions used to construct the deletion mutants are indicated by lines framed by inverted arrow, which represent the positions of primers used for their amplification. The chromosomal organisation, after deletion of SA1665 is shown beneath. The position of the SA1665 transcriptional terminator, which remained intact after SA1665 markerless deletion is indicated (⫯).

A DNA-binding protein purification assay was performed to identify new potential factors involved in the regulation of *mecA*/PBP2a. The *mecA/mecR1 *intergenic DNA region, including the 5' 9 bp of *mecR1 *and the first 52 bp of *mecA*, was used as bait against crude protein extract from strain CHE482. Proteins binding to this DNA fragment were analysed by SDS-PAGE. Even though CHE482 contained BlaI, which is known to bind to the *mec *operator, this band could not be identified on gels due to co-migrating, non-specific bands the same size as BlaI (14.9 KDa) that bound to both the DNA-coated and uncoated control beads. The most prominent protein band of ~16–20 kDa, isolated from DNA-labelled but not from control beads, was identified as the hypothetical protein SA1665 (N315 genome annotation [BA000018]) (Figure 1A). SA1665 encodes a predicted 17-kDa protein with an n-terminal helix-turn-helix (HTH) motif characteristic of DNA-binding transcriptional regulators. The amino acid sequence of SA1665 showed 100% identity amongst *S. aureus *database sequences and 97–98% identity amongst other staphylococci, including *S. haemolyticus*, *S. epidermidis *and *S. saprophyticus*, indicating that SA1665 is highly conserved. Conversely, there were no orfs highly similar to SA1665 found in other bacterial species, with the most similar sequences found in *Bacillus licheniformis *DSM13 and *Desulfitobacterium hafniense *Y51, which shared only 64% and 59% similarity, respectively.

### Electro mobility shift assays (EMSA)

**Figure 2:**
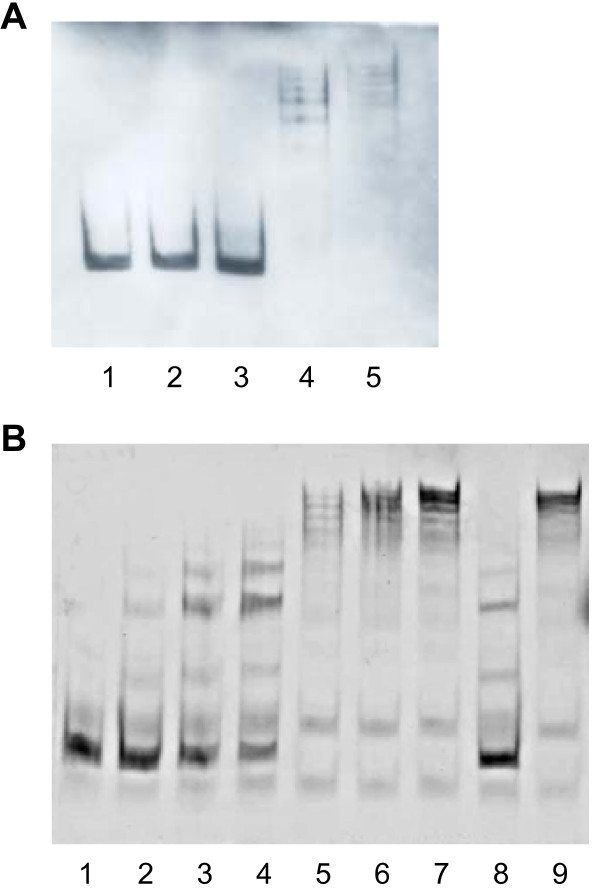
**Electromobility shift of *mec *operator DNA by SA1665**. A, Gel shift using biotinylated DNA (6 ng) and crude protein extracts. Lane 1, DNA only control; lanes 2 and 3, DNA incubated with 200 ng and 500 ng of crude protein extract from *E. coli *BL21 pET28nHis_6_, respectively; lanes 4 and 5, DNA incubated with 200 ng and 500 ng of crude protein extract from *E. coli *BL21 pME20, expressing SA1665, respectively. B, Gel shift of biotinylated DNA (6 ng) with purified SA1665 protein. Lane 1, DNA only control; lanes 2–7 DNA incubated with 10, 40, 75, 150, 200 and 250 ng protein, respectively; lane 8, DNA incubated with 250 ng of protein in the presence of a 130-fold excess of unlabelled specific competitor DNA; lane 9, DNA incubated with 250 ng of protein in the presence of unspecific competitor (herring sperm) DNA.

EMSA was used to confirm binding of SA1665 to the *mec *operator region. Crude protein extracts of *E. coli *strain BL21, carrying the empty plasmid (pET28nHis_6_) or pME20 (pET28nHis_6_-SA1665) which expressed nHis_6_-SA1665 upon induction with IPTG, were incubated with the 161-bp biotinylated-DNA fragment previously used as bait in the DNA-binding protein assay. A band shift was observed with extracts from the strain expressing recombinant nHis_6_-SA1665 but not from the control strain carrying the empty plasmid. Several bands resulted from the shift, which is most likely due to protein oligomerisation (Figure 2A). The specificity of the gel shift was also demonstrated by the addition of increasing concentrations of purified nHis_6_-SA1665 protein to the biotinylated-DNA fragment (Figure 2B). Band-shift of the biotinylated DNA was inhibited in the presence of specific competitor DNA but not by the presence of the non-specific competitor DNA, confirming that nHis_6_-SA1665 had a specific binding affinity for the 161-bp DNA fragment.

### Effect of SA1665 deletion on β-lactam resistance

To analyse the effect of SA1665 inactivation on methicillin resistance, nonpolar markerless deletions of SA1665 (Figure 1B) were constructed in a selection of clinical MRSA isolates, which varied in their genetic background, SCC*mec *type, and *mecA *regulation [24]. Strain CHE482, belongs to clonal complex CC45 and sequence type ST45, and contains a novel SCC*mec *(SCC*mec*_N1 _[23]); while strains ZH37 (CC45/ST45) and ZH73 (CC22/ST22) contain type IV SCC*mecs*. All three of these strains have truncated *mecI*/*mecR1 *regulatory loci but intact BlaI/BlaR1 loci controlling *mecA *expression. Strain ZH44 (CCT8/ST8) contained a type A *mec *complex (*mecI-mecR1-mecA*) within a type II SCC*mec*, and had no β-lactamase locus; so *mecA *was only under the control of its cognate regulators MecI/MecR1.

**Figure 3:**
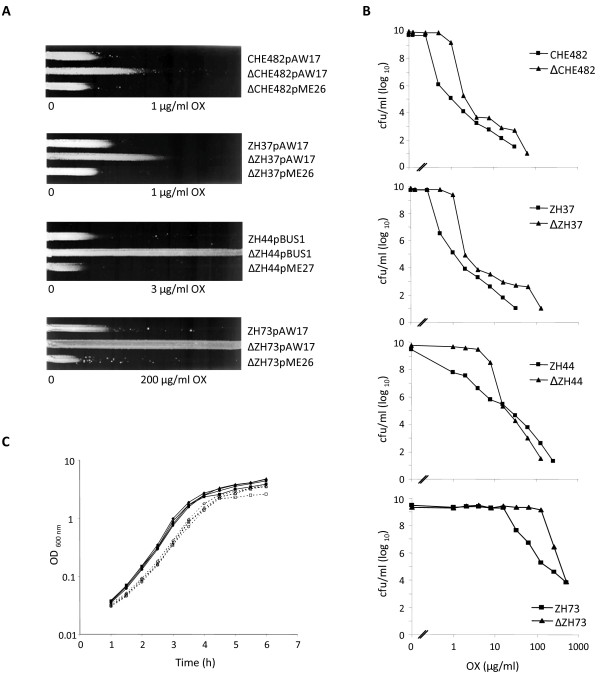
**Effect of SA1665 deletion on oxacillin resistance**. A, Growth of MRSA strains and their SA1665 deletion mutants, containing empty plasmid vector pAW17 or pBUS1, and trans complemented mutants, containing pME26 or pME27, was compared on plates containing appropriate oxacillin gradients, as indicated. Plates were supplemented with either kanamycin (25 μg/ml) or tetracycline (5 μg/ml) to ensure plasmid maintainence. B, Representative population analysis profiles of MRSA strains CHE482, ZH37, ZH44, and ZH73 and their corresponding mutants. Wildtype strains are indicated by squares and mutants by triangles. x- and y-axis show the oxacillin concentrations (μg/ml) and the cfu/ml, respectively. Oxacillin concentrations used were two-fold dilutions ranging from 0.1–256 μg/ml for strains CHE482 and ZH37 and 1–1024 μg/ml for strains ZH44 and ZH73. C, Growth curves of wildtype strains (solid lines, closed symbols) and their corresponding SA1665 mutants (dashed lines, open symbols); CHE482 (diamonds), ZH37 (triangles), ZH44 (circles), ZH73 (squares).

Deletion of SA1665 increased oxacillin resistance in all mutants compared to their corresponding parent strains, as demonstrated on oxacillin gradient plates (Figure 3A); with mutants ΔCHE482 and ΔZH37 approximately doubling in resistance and ΔZH44 and ΔZH73 expressing considerably higher resistance. Population analysis resistance profiles of the mutants showed a distinct shift at the top of the curve, indicating that the higher resistance was due to increased basal oxacillin resistance levels (Figure 3B). Strains CHE482/ΔCHE482 and ZH37/ΔZH37 had very similar resistance profiles, despite having different SCC*mec *elements, suggesting that it was their common clonal background (CC45) that determined their resistance levels and the extent of resistance increase upon SA1665 deletion.

Growth curve analyses showed that deletion of SA1665 slightly reduced the growth rate of all strains tested (Figure 3C). Wild type growth rates were restored upon complementation (data not shown).

### Resistance complementation

Plasmids pME26 and pME27 were constructed for complementation of the deletion mutants. Both plasmids contained the SA1665 orf along with its own promoter and transcriptional terminator. Strains ΔCHE482, ΔZH37, and ΔZH73 were complemented with pME26, and intrinsically kanamycin resistant strain ΔZH44 was complemented with pME27. Wild type-like resistance levels were restored in all mutants by introduction of the complementing plasmids, as shown by gradient plates (Figure 3A).

### Transcriptional analyses

**Figure 4:**
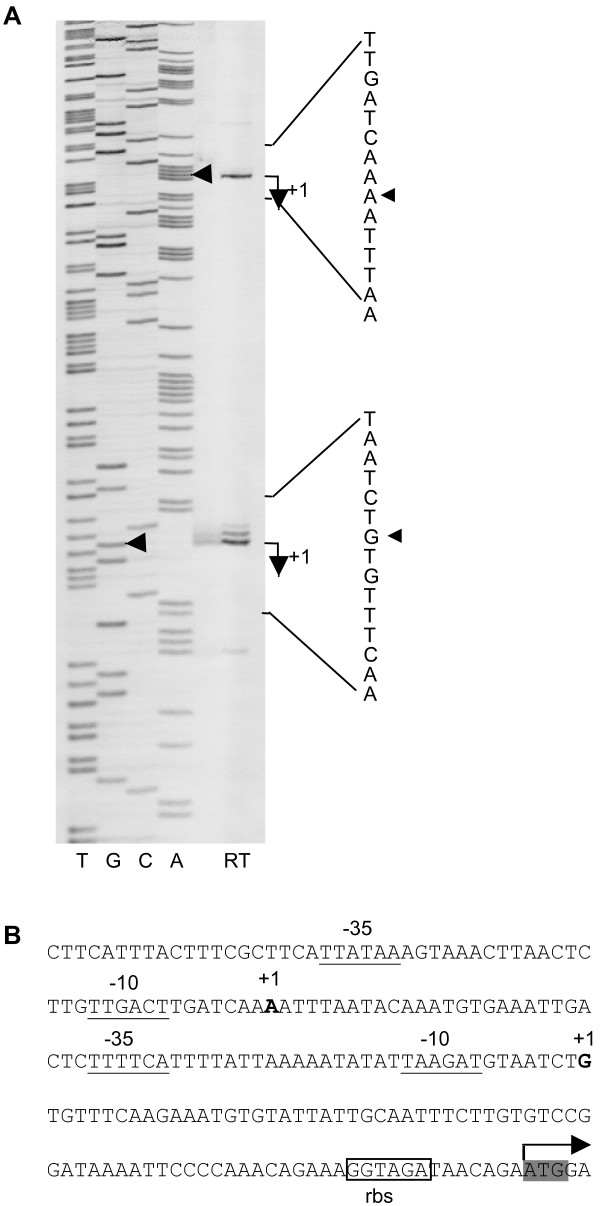
**Primer extension analysis of SA1665**. A, Lanes A, C, G, T show the dideoxy-terminator sequencing ladder and lane RT the reverse transcription products obtained using primer me97. Two potential transcriptional start sites (TSS) were identified, as indicated by arrows (◀). B, Sequence of the SA1665 promoter region. TSS (+1) are shown in bold, putative -10 and -35 promoter sequences are underlined, the predicted ribosome binding site (rbs) is framed and the translational start (ATG) of SA1665 is highlighted in grey.

Primer extension, using the 5'-biotinylated primer me97, identified two potential SA1665 transcriptional start sites (TSS), 76-nt and 139-nt upstream of the SA1665 ATG start codon (Figure 4A). Predicted σ^A ^promoter consensus -10/-35 box sequences were located upstream of both TSS (Figure 4B). Identical TSS were also identified using the downstream primer me98 (data not shown).

**Figure 5:**
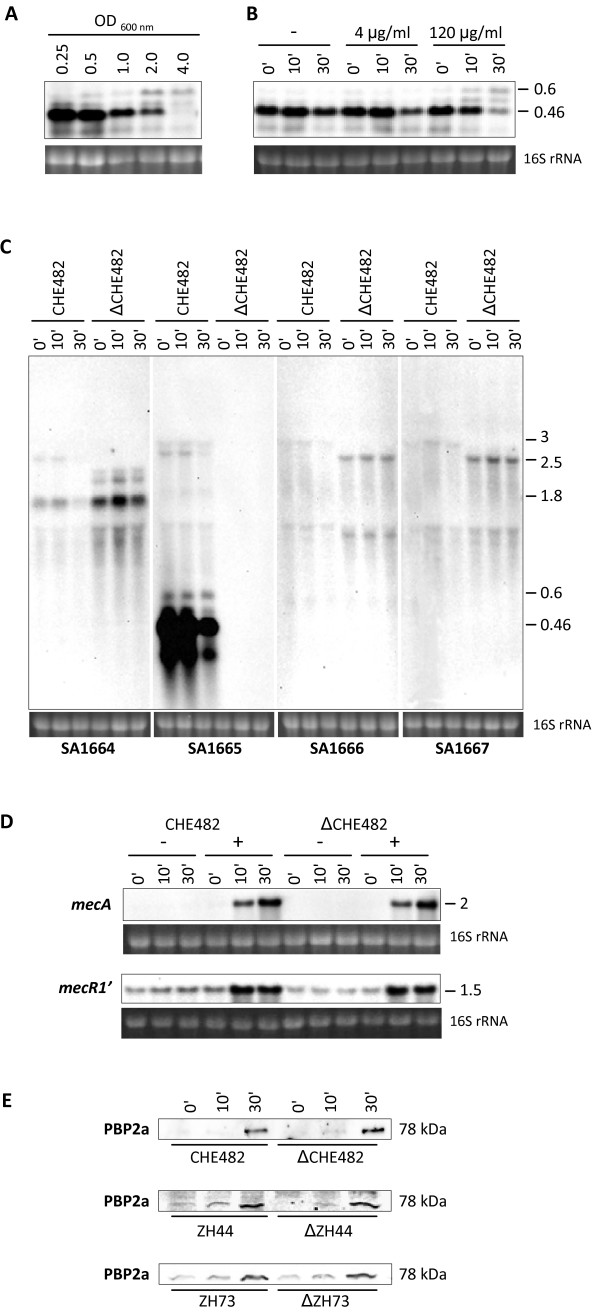
**Northern and Western blot analyses**. A, Transcription of SA1665 over growth in CHE482, with RNA harvested at the OD_600 nm _values indicated. B, Transcription of SA1665 from CHE482 grown to OD_600 nm _0.25 and either left uninduced (-) or induced with either 4 or 120 μg/ml of cefoxitin fo 0', 10' and 30'. C, Transcriptional profiles of SA1664, SA1665, SA1666 and SA1667 in CHE482 and ΔCHE482, grown to OD_600 nm _0.25 and either uninduced or induced with cefoxitin 4 μg/ml for 0', 10' or 30'. Approximate sizes of transcripts, in kb, are indicated on the right of the blots. D, Transcription of *mecA *and *mecR1 *in CHE482 and ΔCHE482, grown to OD 0.25 and either left uninduced or induced with cefoxitin (4 μg/ml) and sampled after 0', 10' and 30'. Ethidium bromide stained 16S rRNA bands from all Northern gels are shown as a comparative indication of RNA loading. E, Western blots showing amounts of PBP2a in ZH44 and ZH73 and their respective SA1665 deletion mutants, before (0') and after induction with 4 μg/ml of cefoxitin for 10' and 30'.

Northern blot analysis was used to investigate SA1665 expression and the influence of SA1665 deletion on *mecA *and *mecR1 *transcription. RNA samples taken from different time points over the growth curve of CHE482 showed that SA1665 was expressed strongly in early exponential phase at OD_600 nm _0.25 and 0.5, then transcript levels decreased and were almost undetectable in early stationary phase at OD_600 nm _4.0 (Figure 5A). In addition to the main transcript of ~0.46 kb, a weaker, larger transcript of ~0.6 kb was also visible, especially at later growth stages. Figure 5B shows the transcriptional behaviour of SA1665 when CHE482 cells were challenged with sub-inhibitory (4 μg/ml) and inhibitory (120 μg/ml) concentrations of cefoxitin. These results showed that low levels of cefoxitin, such as those used to induce *mecA*/*mecR1 *transcription, appeared to slightly decrease SA1665 transcription after 30 min exposure, while larger, inhibitory concentrations caused even more significant alterations in the SA1665 transcriptional profile, making it similar to that normally seen in stationary phase growth. These results indicate that transcription of SA1665 may respond in some way to cell wall stress, rather than in direct response to the presence of β-lactams. This observation is based on relatively subtle changes in SA1665 transcription, especially at low concentrations of cefoxitin such as those required for *mecA*/*mecR1 *induction. Since deletion of SA1665 has been shown to increase β-lactam resistance, reduced SA1665 transcription in the presence of β-lactams may also provide some protection against β-lactam exposure.

Northerns also showed that, as expected, the SA1665 transcripts were absent from the deletion mutant (Figure 5C), and additional experiments demonstrated that wild type SA1665 transcription patterns were restored by complementation of ΔCHE482 with pME26 (data not shown). The effects of SA1665 deletion on directly up- and down-stream genes were also investigated. Northern blots of the neighbouring genes SA1664, SA1666 and SA1667, showed that expression of all three genes was very weak compared to that of SA1665. A weak transcript of about 3 kb was present in hybridizations probed with orfs SA1665-SA1667. This band decreased in size in the SA1665 mutant when probed with SA1666 and SA1667. One of the transcripts hybridising to the SA1664 probe also decreased in size by ~0.5 kb in the SA1665 mutant, suggesting that SA1665 was present on several transcripts of different lengths, including a high abundance monocistronic transcript and low abundance polycistronic transcripts (Figure 5C). Transcript abundance of both the upstream SA1666-SA1667 operon and the downstream SA1664-specific transcript all appeared to increase slightly in ΔCHE482. The significance of these subtle increases in transcription are unknown, however, polar effects from SA1665 deletion seem unlikely, based on the facts that all genes were still transcribed, their transcription levels all remained extremely low and the transcriptional terminator of SA1665 remained intact in the deletion mutant (Figure 1B).

Expression of *mecR1 *and *mecA *were analysed from RNA of uninduced and induced cultures of CHE482 and ΔCHE482. Cells were induced at OD_600 nm _0.25 (Figure 5D) and 1.0 (data not shown) with sub-inhibitory concentrations of cefoxitin, to relieve BlaI-repression of *mecA*. *mecR1*, although truncated in CHE482, was still transcribed and had the same expression pattern as *mecA*, as both became derepressed over time and had the highest transcript levels after 30 min of induction. In the mutant ΔCHE482, transcripts of both *mecA *and *mecR1' *were unaffected by SA1665 deletion, indicating that SA1665 had no influence on their expression at either OD 0.25 (Figure 5D) or OD 1.0 (data not shown). SA1665 deletion also had no effect on *mecA *transcription or induction in strains ZH37, ZH44 and ZH73 (data not shown).

### Western blot analysis

Mutants of CHE482 and of ZH44 and ZH73, which had the largest differences in oxacillin resistance levels, were analysed by Western blot analysis to determine if SA1665 affected production of PBP2a from *mecA*. As shown in Figure 5E, all pairs of wild type and mutant strains had similar amounts of PBP2a present both before and after induction with cefoxitin, indicating that SA1665 deletion did not alter amounts of PBP2a produced. Therefore it seems that SA1665 exerts no direct control over *mecA *or PBP2a expression.

## Discussion

Methicillin resistance in MRSA is primarily dependent on the presence of the *mecA *gene, however, resistance levels are generally governed by strain-specific factors including *mecA *regulatory elements and other chromosomal *fem/aux *factors which either enhance or repress the expression of resistance. For instance, the very low-level methicillin resistance of the Zurich drug clone CHE482, was shown to be controlled by its genetic background [12] suggesting that it either contained or lacked certain *fem/aux *factors involved in controlling resistance expression. Many of the currently known *fem/aux *factors are directly or indirectly involved in cell wall synthesis and turnover, or envelope biogenesis, however there still remain factors of unknown function. Most of the currently known *fem/aux *factors reduce methicillin resistance levels when inactivated. A few genes, such as *lytH, dlt*, *norG*, *sarV *and *cidA *increase resistance levels upon inactivation or mutation. All of these genes, except *norG*, which is an efflux pump regulator, play a role in either autolysis or are important for cell physiology and growth [25-30]. Other genes increase β-lactam resistance upon overexpression, such as *hmrA *coding for a putative amidohydrolase, *hmrB *coding for a putative acyl carrier protein [31], or the NorG-controlled *abcA *multidrug efflux pump [28].

SA1665, a predicted DNA-binding transcriptional regulator, was found to bind to a DNA fragment containing the *mecA *promoter region. However, although this protein shifted the *mecA *operator/5' coding sequence, it did not appear to directly control *mecA *or *mecR1 *transcription or PBP2a production. Therefore its binding to the *mecA *region may have no specific regulatory function. Such interactions have been noted before, such as the HTH protein NorG, which was shown to bind specifically to *norA*, *norB *and *norC *promoters, but only transcription of *norB *was increased when NorG was overexpressed [28]. We have to postulate therefore that SA1665 may modulate β-lactam resistance in a *mecA*-independent manner, by controlling cellular functions affecting resistance levels. Experiments to determine the SA1665 regulon are ongoing. The impact of deleting SA1665 in MRSA was extremely strain specific, underlining the importance of the genetic background in governing the final methicillin resistance levels of MRSA, and demonstrating the large genomic variability between different strain lineages.

## Conclusion

SA1665 is a previously uncharacterised DNA-binding protein that has a negative effect on β-lactam resistance in MRSA. The SA1665 protein was identified in a DNA-binding protein purification assay, in which it bound to a DNA fragment covering the *mec *operator region. However, while nonpolar deletion of SA1665 was shown to increase oxacillin resistance levels in several heterogeneously resistant MRSA, its deletion had no effect on *mecA *transcription or PBP2a production. Therefore the negative impact of SA1665 on methicillin resistance is most likely to be through the regulation of other chromosomal factors or cellular functions required for methicllin resistance.

## Methods

### Strains and growth conditions

Strains and plasmids used in this study are listed in Table 1. Clinical isolates are from the IMM collection in Zurich, Switzerland. Strains were grown at 37°C in Luria Bertani (LB) broth, shaking at 180 rpm, or on LB agar. Media were supplemented with the following antibiotics when appropriate: 25 or 50 μg/ml kanamycin, 10 μg/ml chloramphenicol, 5 or 10 μg/ml tetracycline, 100 μg/ml ampicillin. Concentrations of cefoxitin used for transcriptional induction were either sub-inhibitory (4 μg/ml) or inhibitory (120 μg/ml).

**Table 1:** Strains and plasmids used in this study.

Strain/plasmid	Relevant genotype^*a*^	Reference/source
*S. aureus*		
CHE482	clinical MRSA isolate, CC45/ST45, SCC*mec*_N1_, *blaZ *(pBla)	[23,24]
ΔCHE482	CHE482 ΔSA1665	this study
ZH37	clinical MRSA isolate, CC45/ST45, SCC*mec *type IV, *blaZ*	[24]
ΔZH37	ZH37 ΔSA1665	this study
ZH44	clinical MRSA isolate, CCT8/ST8, SCC*mec *type II, *aac-aph*	[24]
ΔZH44	ZH44 ΔSA1665	this study
ZH73	clinical MRSA isolate, CC22/ST22, SCC*mec *type IV, *blaZ*	[24]
ΔZH73	ZH73 ΔSA1665	this study
RN4220	NCTC8325-4, restriction negative	[38]
*E. coli*		
DH5α	restriction-negative strain for cloning	Invitrogen
BL21 (DE3)	F^- ^*omp*T *hsd*S_B_(r_B_^-^m_B_^-^) *gal dcm *(DE3)	Novagen
Plasmids		
pBUS1	*S. aureus-E. coli *shuttle vector, *tetL*	[37]
pAW17	*S. aureus-E. coli *shuttle vector, *aac-aph*	[37]
pKOR1	*S. aureus-E. coli *shuttle vector, *cat, bla*	[34]
pME17	pKOR1-SA1664/SA1666, *cat*	this study
pET28nHis_6_	*E. coli *protein expression vector, with n-terminal His_6 _tag, *aac-aph*	D. Frey, unpublished
pME20	pET28nHis_6_-SA1665, *aac-aph*	this study
pME26	pAW17-SA1665 and 700 bp up- and 380 down-stream, *aac-aph*	this study
pME27	pBUS1-SA1665 and 700 bp up- and 380 bp down-stream, *tetL*	this study

^*a*^Abbreviations: CC, clonal complex; ST, sequence type.

### Susceptibility testing

Oxacillin resistance levels were compared by swabbing 0.5 McFarland cell suspensions across agar plates containing appropriate concentration gradients of oxacillin. For population analysis profiles, appropriate dilutions of an overnight culture, ranging from 10^0 ^to 10^8^, were plated on increasing concentrations of oxacillin. Plates were incubated at 35°C and colony forming units per ml (cfu/ml) were determined after 48 h.

### Binding-protein purification

Crude protein extracts were isolated from CHE482, grown under normal culture conditions until OD_600 nm _1.5. Cells were harvested, resuspended in PBS (pH 7.4) and mechanically lysed using Lysing Matrix B (BIO 101 Systems) tubes and a FastPrep FP120 (BIO 101 Systems). Suspensions were clarified by centrifugation and supernatants, containing soluble cytoplasmic proteins, were transferred to Amicon Ultra centrifugal filter devices (Millipore) with a pore cut-off size of 10 kDa. Proteins were then washed and concentrated in 1× binding buffer (10 mM Tris-HCl, pH 7.5, 1 mM EDTA, and 1 mM DTT, 0.5 M NaCl). Protein concentrations were measured by Bradford assay (BioRad Laboratories GmbH) [32]. Primer pair me36F/me36Rbiot (Table 2) were used to amplify a biotinylated *mecA *promoter/operator fragment, which was bound to streptavidin coated magnetic beads (Dynabeads M-280 Streptavidin, DYNAL BIOTECH) according to the manufacturer's instructions. Binding reactions, containing DNA-coated beads mixed with 100 μg of crude protein extract in 1× protein binding buffer (20 mM Hepes, pH 7.6, 1 mM EDTA, 10 mM (NH_4_)_2_SO_4_, 1 mM DTT, 0.2% Tween 20 (w/v), 30 mM KCl), 0.02 μg/μl poly d(I-C) and 2 ng/μl poly L-lysine, were incubated at room temperature for 30 min with constant rotation. Beads were then washed and binding-proteins eluted in elution buffer (1× protein binding buffer containing 2 M KCl). Eluted proteins were dialysed against water, concentrated by evaporation, and run on 15% SDS polyacrylamide gels. Gels were silver stained using the Protein Silver Staining kit (Amersham Biosciences AB) without the addition of glutaraldehyde. Protein bands were excised from gels and analysed by mass spectrometry (LC/ESI/MS/MS) at the Functional Genomics Centre, Zurich. The SA1665 protein sequence [BAB42933] was analysed by Blast search http://www.ncbi.nlm.nih.gov/BLAST and motif scan http://myhits.isb-sib.ch/cgi-bin/motif_scan.

**Table 2:** Oligonucleotide primers used in this study.

Primer name	Nucleotide sequence (5'-3')^*a*^	Reference
Markerless deletion construction		
me62attB1	GGGGACAAGTTTGTACAAAAAAGCAGGCTCCACTGGCTTATTCGCTTGA	This study
me51BamHI	ATTAGGATCCTTAGTACATATCTAGGCCTA	This study
me62BamHI	ATTAGGATCCACTCTGTCTATCCATTCTGT	This study
me62attB2	GGGGACCACTTTGTACAAGAAAGCTGGGTTGTGCGACAAGGATTGCGA	This study
Cloning		
me94BamHI	ATTAGGATCCTCTTCAATCACTTGGCCAAT	This study
me94Asp718	ATTAGGTACCAAGGTGCTGATGGTTATGAA	This study
me65BamHI	ATTAGGATCCGATAGACAGAGTTTTACAGA	This study
me65XhoI	ATTACTCGAGGATATGTACTAATTCTTCTT	This study
Protein-DNA binding and EMSA		
me36F	TGATAACACCTTCTACACCT	This study
me36Rbiot	BIOT-AACCCGACAACTACAACTAT	This study
me36R	AACCCGACAACTACAACTAT	This study
Primer extension		
me97	BIOT-ACTCTGTCTATCCATTCTGT	This study
me98	BIOT-CAGCCTCTATACGAACCATT	This study
me52F	CCACTGGCTTATTCGCTTGA	This study
me52R	TGTGCGACAAGGATTGCGAT	This study
Gene/transcript detection		
mecAP4	TCCAGATTACAACTTCACCAGG	[42]
mecAP7	CCACTTCATATCTTGTAACG	[42]
SA1665F	TTCGTATAGAGGCTGGTTAG	This study
SA1665R	AATTGGTTGGTTATCTGGAT	This study
mecR1F	TGACACGACTTCTTCGGTTA	This study
mecR1R	AACGTATATGTTCATGGCGA	This study
SA1664F	TCAGCATGTAGATAACGCAA	This study
SA1664R	ATGTCACAATTGTTCTTGCT	This study
SA1666F	GACCATTATATTGTGCGACA	This study
SA1666R	TTGTGCCTTAGGATGTATCA	This study
SA1667F	TTGTGCCTTAGGATGTATCA	This study
SA1667R	TAATACCGTGTGATGAAGCT	This study

^*a*^Restriction sites are underlined; BIOT: end-labelled with biotin.

### Expression of recombinant SA1665 protein

SA1665 was amplified using primer pair me65BamHI/me65XhoI (Table 2) and cloned in-frame into pET28nHis_6 _(unpublished, D. Frey). The resulting plasmid, pME20, was transformed into *E. coli *BL21 for expression of recombinant nHis_6_-SA1665 protein. To maximise the abundance of soluble protein produced, cultures were grown in osmotic shock medium at 37°C (1 g/l NaCl, 16 g/l tryptone, 10 g/l yeast, 1 M sorbitol, 10 mM betaine, modified from [33]) to an OD_600 nm _of 0.5, cooled briefly on ice, then induced by adding 100 μM IPTG and growing overnight at 22°C. Crude soluble proteins were extracted using CelLyticB 2× cell lysis reagent (SIGMA). HIS-Select Cobalt Affinity Gel (SIGMA) was used to purify recombinant nHis_6_-SA1665 according to the manufacturer's instructions.

### Electro mobility shift assay

For gel shift assays, 6 ng aliquots of the biotinylated-DNA fragment used for binding-protein purification were incubated with 0–250 ng of purified nHis_6_-SA1665 protein in 1× binding buffer (20 mM Hepes pH 7.6, 1 mM EDTA, 10 mM (NH_4_)_2_SO_4_, 1 mM DTT, 0.2% Tween 20 (w/v), 30 mM KCl) containing 0.05 μg/μl poly d(I-C) (Roche) and 5 ng/μl poly L-lysine (Roche). For control binding reactions, 130 × unlabelled *mec *operator DNA (amplified using primers me36F/me36R, Table 2) was used as a specific binding competitor and 6 ng of herring sperm DNA was used as unspecific competitor DNA. Binding was carried out at 22°C for 30 min. Samples were run on 6% native polyacrylamide gels, contact blotted onto positively charged nylon membrane and detected with the Biotin Chromogenic Detection Kit (Fermentas).

### Primer extension

RNA was extracted from CHE482 cultures that were grown to OD_600 nm _0.5, as previously described [12]. Primer extension reactions were performed using 20 μg of total RNA and 3 pmol of the 5'-biotin-labelled primers me97 and me98 (Table 2) using Superscript II reverse transcriptase (Invitrogen), according to the manufacturers instructions. Sequencing reactions were performed using the Thermo Sequenase cycle sequencing kit (U.S. Biochemicals). The Biotin Chromogenic Detection Kit (Fermentas) was used for biotin detection.

### Markerless deletion of SA1665

In frame markerless deletions of SA1665, from the chromosomes of CHE482, ZH37, ZH44, and ZH73, were constructed using the pKOR1 allelic replacement system, as described by Bae et al. [34]. Primer pairs used to amplify the DNA fragments flanking SA1665, for recombination into pKOR1 were: me62attB1/me51BamHI and me62BamHI/me62attB2 (Table 2). All deletion mutants were confirmed by nucleotide sequencing over the deleted region, as well as by Southern blot analysis [35] and pulsed field gel electrophoresis (PFGE) [36].

### Cloning of SA1665 for complementation

A 1533-bp DNA fragment, containing SA1665 together with 690-bp of upstream and 379-bp of downstream DNA, was amplified from strain CHE482 using primers me94BamHI/me94Asp718 (Table 2) and cloned into the *E. coli/S. aureus *shuttle vectors pAW17 and pBUS1 [37], creating the complementing plasmids pME26 and pME27, respectively. Plasmids were electroporated into RN4220 [38] and then transduced into different strains using phage 80α.

### Northern blot analysis

Strains were grown overnight in LB (Difco), diluted 1:200 and grown for another 3 h. This preculture was used to inoculate 150 ml (1:1000) of fresh prewarmed LB. Cells were then grown to OD_600 nm _0.25 or 1.0 and either left uninduced or induced with cefoxitin 4 or 120 μg/ml. Cultures were sampled from both uninduced and induced cells at time point 0' before induction and at 10' and 30' (min) after induction. To monitor SA1665 expression over growth, separate cultures were also sampled at different growth stages corresponding to OD_600 nm _0.25, 0.5, 1, 2, and 4. Total RNA was extracted as described by Cheung et al. [39]. RNA samples (10 μg) were separated in a 1.5% agarose-20 mM guanidine thiocyanate gel in 1× TBE running buffer [40], then transferred and detected as described previously [41]. Digoxigenin (DIG) labelled-probes were amplified using the PCR DIG Probe synthesis kit (Roche). Table 2 contains the list of primer pairs used for the amplification of SA1664, SA1665, SA1666, SA1667, *mecR1 *and *mecA *[42] probes. All Northern's were repeated at least two times, using independently isolated RNA samples.

### Western blot analysis

Cells were cultured, as described for Northern blot analysis, to OD_600 nm _1.0, then induced with cefoxitin 4 μg/ml. Samples were collected at time 0 (before induction), 10 and 30 min (after induction). Cells were harvested by centrifugation, resuspended in PBS pH 7.4 containing DNase, lysostaphin and lysozyme (150 μg/ml of each) and incubated for 1 h at 37°C. Suspensions were then sonicated and protein aliquots (15 μg) were separated on 7.5% SDS-polyacrylamide gels, blotted onto nitrocellulose membranes (Hybond) and stained with Ponceau to confirm equal protein loading. PBP2a detection was performed using monoclonal PBP2a antibody (1:20000) from the MRSA-screen kit (Denka Seiken).

## Authors' contributions

ME carried out molecular genetic and microbiological studies and drafted the manuscript. BB participated in the design of the study and helped to draft the manuscript. NM participated in the design and coordination of the study, carried out molecular biological studies and helped to draft the manuscript. All authors read and approved the final manuscript.
